# Unraveling the role of HIF-1 in peripheral blood mononuclear cells from older patients with Alzheimer’s disease

**DOI:** 10.3389/fnagi.2026.1831919

**Published:** 2026-06-03

**Authors:** Beatrice Arosio, Evelyn Ferri, Paolo Dionigi Rossi, Jacopo Aiello, Simone Caponetto, Fabiola Olivieri, Chiara Fenoglio, Daniela Galimberti, Tiziano Angelo Lucchi, Nicola Montano

**Affiliations:** 1 Department of Clinical Sciences and Community Health, Dipartimento di Eccellenza 2023–2027, University of Milan, Milan, Italy; 2 Fondazione IRCCS Ca’ Granda Ospedale Maggiore Policlinico, Milan, Italy; 3 San Leopoldo Mandic Hospital, Merate, Italy; 4 Department of Clinical and Molecular Sciences, Università Politecnica delle Marche, Ancona, Italy; 5 Advanced Technology Center for Aging Research, IRCCS INRCA, Ancona, Italy; 6 Department of Biomedical, Surgical and Dental Sciences, University of Milan, Milan, Italy

**Keywords:** Alzheimer’s disease, hypoxia response elements, hypoxia-inducible factor 1, neuroinflammation, peripheral blood mononuclear cells

## Abstract

**Introduction:**

Cerebrovascular damage is increasingly recognized as an early event in the dementia continuum, occurring before typical Alzheimer’s disease (AD) pathological changes. Hypoxia-inducible factor 1 (HIF-1) is a transcription factor composed of HIF-1α and HIF-1β subunits which, under hypoxic conditions, dimerize and activate hypoxia response element (HRE)-containing genes. HIF-1α has been reported to be implicated in neuroinflammation, a key feature of AD.

**Methods:**

This study evaluated HIF1A and its negative regulator HIF1AN gene expression in peripheral blood mononuclear cells (PBMCs) from 308 cognitively healthy older individuals (controls) and 83 AD patients, and their associations with gene expression of HRE-containing inflammatory genes in PBMCs and corresponding protein concentrations in plasma.

**Results:**

Peripheral blood mononuclear cells from AD patients showed lower gene expression of both HIF1A and HIF1AN compared with controls, and this reduction was associated with higher odds of AD. In the overall cohort, after adjustment for age, sex, Apolipoprotein E ε4 status, and diagnosis, HIF1A gene expression was positively associated with interleukin (IL)-6, IL-10, tumor necrosis factor-α (TNF-α), IL-1B, and triggering receptor expressed on myeloid cells-1 (TREM-1) gene expression, whereas HIF1AN gene expression was negatively associated with IL-6, IL-1B, and TREM-1 gene expression. Furthermore, HIF1A gene expression was positively associated with plasma IL-1β and soluble TREM-1 concentrations, while HIF1AN gene expression was negatively associated with IL-10 concentrations.

**Discussion:**

Overall, these findings support the use of peripheral cells to investigate HIF-1 pathway dysregulation in AD and suggest that altered HIF-1α signaling may reflect impaired cellular responsiveness linked to neuroinflammatory processes.

## 1 Introduction

The last decades have witnessed a dramatic demographic transition worldwide due to the increase in average life expectancy, which has resulted in a significant increase in the number of old and very old people and consequently age-related diseases (Jones and Dolsten, 2024). Dementia is one of the major age-related diseases characterized by cognitive decline, behavioral disorders and functional impairment accompanied by varying degrees of disability (World Health Organization, 2025).

Alzheimer’s disease (AD) represents the most prevalent form of dementia (60%–70% of cases worldwide) (World Health Organization, 2025). Although the pathogenesis of AD remains unclear, its defining neuropathological changes can be detected many years before the onset of the disease itself. Interestingly, a new hypothetical model of AD dynamics suggests that damage to the cerebral vasculature is an early process in the dementia continuum antecedent to typical AD changes (e.g., neuroinflammation and neurodegeneration) (Sargurupremraj et al., 2020; Rajeev et al., 2022). Cerebrovascular dysfunction is closely associated with impaired cerebral blood flow and oxygen delivery, leading to a hypoxic-like environment in the brain. In addition to disrupting oxygen metabolism, hypoxia promotes reactive oxygen species production and inflammatory responses (Chen et al., 2018), thereby contributing to AD pathogenesis and potentially accelerating disease progression (Sun et al., 2006; Liu et al., 2016).

Hypoxia-inducible factor 1 (HIF-1) is a transcription factor consisting of two subunits, HIF-1α and HIF-1β, which are crucial for maintaining cellular homeostasis under hypoxic conditions. HIF-1α is ubiquitously expressed and negatively modulated under normoxic conditions, while HIF-1β is constitutively expressed acting as a co-activator in several transcriptional processes (Zhang et al., 2025). The regulation of HIF-1α occurs through two main oxygen-dependent mechanisms. First, its stability is controlled by proteasomal degradation following the hydroxylation of two highly conserved prolyl residues (Pro402 and Pro564) by prolyl hydroxylase domain (PHD) enzymes (Strowitzki et al., 2019), which enables recognition and polyubiquitination by an E3 ubiquitin ligase complex containing the von Hippel–Lindau (VHL) protein (Yfantis et al., 2023). The second mechanism involves the transcriptional inactivation of HIF-1α by factor-inhibiting HIF (FIH), which hydroxylates a conserved asparagine residue (Asn803) within the C-terminal transactivation domain of HIF-1α, thereby preventing the recruitment of transcriptional coactivators such as p300/CBP and ultimately inhibiting its transcriptional activity on target genes (Volkova et al., 2022). Under hypoxic conditions, the negative modulation mechanisms are less efficient, and the stability of this factor increases allowing the translocation of HIF-1α to the nucleus, where it heterodimerizes with HIF-1β to form the HIF-1 active transcription complex driving the expression of target genes with the hypoxia-response elements (HREs) in their promoters (Yfantis et al., 2023). In recent years, the pathophysiological relevance of FIH-mediated regulation of HIF-1α has been increasingly demonstrated across a range of disease contexts, including cancer (Schützhold et al., 2022; García-del Río et al., 2023; Bargiela et al., 2024; Ma et al., 2024), fibrosis (Brereton et al., 2022), inflammation (Schützhold et al., 2022; Ting et al., 2024), and metabolic dysfunction (Ting et al., 2024; Wu et al., 2024; Table 1).

**TABLE 1:** Recent functional studies on FIH-mediated regulation of HIF-1α signaling across pathological contexts.

Reference	Clinical condition	Sample	Main findings
(Schützhold et al., 2022)	Chronic colitis/colorectal cancer	Murine colon epithelium	FIH deficiency in colon epithelium attenuates chronic colitis severity and reduces macrophage infiltration through downregulation of HIF-1α-dependent inflammatory genes.
(Brereton et al., 2022)	Idiopathic pulmonary fibrosis	Human idiopathic pulmonary fibrosis fibroblasts	Reduced FIH activity promotes pseudo-hypoxic HIF-1α signaling in fibroblasts, leading to altered collagen organization and pathological lung remodeling in idiopathic pulmonary fibrosis.
(García-del Río et al., 2023)	Lung cancer	Human lung cancer tissue and murine cancer models	FIH deficiency enhances HIF-1α-driven glycolytic reprogramming, impairing tumour cell proliferation, survival, and metabolic adaptation to hypoxia.
(Ma et al., 2024)	B cell lymphoma	Murine myeloid cells	FIH deficiency in myeloid cells promotes M2-like macrophage polarization and remodels the tumour immune microenvironment, thereby enhancing B-cell lymphomagenesis through HIF-1α-dependent and -independent mechanisms.
(Wu et al., 2024)	Obesity/metabolic dysfunction	Murine obesity model	Pharmacological inhibition of FIH enhances HIF-1α-dependent metabolic reprogramming, improving systemic metabolic activity and alleviating obesity-associated dysfunction.
(Bargiela et al., 2024)	Melanoma	Murine CD8+ T cells	FIH deficiency in CD8+ T cell promotes effector differentiation and enhances anti-tumour activity through oxygen-sensitive HIF-dependent mechanisms, improving T-cell-mediated tumour control.
(Ting et al., 2024)	Atherosclerosis	Murine macrophage model	Cholesterol loading enhances PHD and FIH activity in LPS-stimulated macrophages, suppressing HIF-1α stabilization and activity, thereby reducing glycolytic metabolism and attenuating inflammatory responses.

FIH, factor-inhibiting hypoxia-inducible factor 1; HIF-1α, hypoxia-inducible factor 1α; PHD, prolyl hydroxylase domain enzymes; LPS, lipopolysaccharide.

Using genome-wide chromatin immunoprecipitation, hundreds of genes have been identified that are altered in a HIF-1-dependent manner in response to hypoxia (Lombardi et al., 2022), through mechanisms acting both directly on HREs and indirectly via the regulation of transcriptional repressors and microRNAs (Gao et al., 2021; Tarhriz et al., 2023; Benak et al., 2025; Shi and Gilkes, 2025). In the context of AD, HIF-1α has been increasingly recognized as a key mediator of neuroinflammation: its activation in glial cells can upregulate pro-inflammatory cytokines, including interleukin-1β (IL-1β), interleukin-6 (IL-6) and tumor necrosis factor-α (TNF-α), thereby exacerbating neuronal damage (Porel et al., 2025), while dysregulated HIF-1α signaling may further contribute to altered microglial activation and astrocyte reactivity (Porel et al., 2025).

Therefore, in light of these premises and considering that AD is increasingly recognized not merely as a brain-confined disease but also as a systemic disorder with manifestations extending beyond the central nervous system (Morris et al., 2014), we designed this study to pursue the following two main objectives, using peripheral blood mononuclear cells (PBMCs) as a minimally invasive peripheral model to investigate AD-related pathophysiological mechanisms (Arosio et al., 2014; Phongpreecha et al., 2020; Costa et al., 2022; Dover et al., 2024).

First, given the established role of FIH as a key regulator of HIF-1α transcriptional activity, we aimed to compare the gene expression of HIF1A (encoding HIF-1α) and HIF1AN (encoding FIH), in PBMCs from older individuals without cognitive decline and from AD patients. Second, we aimed to investigate the association of HIF1A and HIF1AN gene expression with the expression of genes containing HREs and involved in neuroinflammatory processes [IL-6, interleukin-10 (IL-10), TNF-α, IL-1β, and triggering receptor expressed on myeloid cells-1 (TREM-1)] from PBMCs of the same samples, as well as with their corresponding protein concentrations in blood.

## 2 Materials and methods

### 2.1 Study design

A cohort of 391 community-dwelling older adults (65–93 years old) was enrolled at the Geriatric Unit of the Fondazione IRCCS Ca’ Granda Ospedale Maggiore Policlinico (Milan, Italy) from March 2009 to April 2022. At enrolment, a multidimensional geriatric assessment was conducted to collect information on the medical history as well as cognitive and functional status (i.e., Mini-Mental State Examination, MMSE; Clock Drawing Test, CDT; Activity of Daily Living, ADL, Geriatric Depression Scale, GDS). The neurocognitive assessment was improved by a battery of neuropsychological tests (i.e., Trail Making Test, Verbal Fluency Test, Digit Span Forward and Backward Tests, Verbal Learning Tests, Token Test, Rey’s Figure Copy and Delayed Recall, Raven Colored Progressive Matrices). Information was recorded in the “Geriatrics Unit Data Collection Register” (REGE, protocol number 223248).

This study included 83 patients with AD diagnosis (Dubois et al., 2014). The 308 subjects without cognitive decline (controls) had MMSE score > 24, neuropsychological test negative for dementia, and absence of neurological or psychiatric disorders.

Patients with type 2 diabetes mellitus, as well as individuals receiving lipid-lowering drugs or angiotensin II receptor blockers, were excluded due to the known potential of these conditions and treatments to modulate HIF-1α signaling. In addition, subjects with acute inflammatory states or chronic immune-mediated inflammatory diseases (i.e., Crohn’s disease, ulcerative colitis, rheumatoid arthritis, psoriasis, systemic lupus erythematosus, asthma, and multiple sclerosis) were also excluded, given their established impact on systemic inflammation.

All analyses were performed on biological samples stored within a bio-repository and collected at the time of enrolment under standardized protocols. Only participants with appropriately preserved specimens meeting quality criteria were included in the study.

All participants gave their consent, personally or through their legal guardian if the person did not possess the mental faculties to make an autonomous decision, to the use of their data for research purposes, suitably anonymized. The study protocol received approval from the Ethical Committee of the Fondazione IRCCS Ca’ Granda Ospedale Maggiore Policlinico (REGE 2.0, protocol number 1696).

### 2.2 Sample analysis

At recruitment, 6-h fasting blood samples were collected in Ethylenediaminetetraacetic acid (EDTA) tubes between 8 and 9 a.m. EDTA-blood was centrifuged at 1200 *g* for 15 min at room temperature.

#### 2.2.1 Protein concentration in plasma

Platelet-free plasma samples were stored at temperatures below −80 °C and thawed immediately prior to analysis. Plasma concentrations of IL-6, IL-10, TNF-α, IL-1β, and sTREM-1 were quantified using Human Simple Plex assays (ProteinSimple, CA, USA) on the Ella platform (ProteinSimple, CA, USA). Instrument calibration was carried out using the factory-generated standard curve embedded in each cartridge, and plasma samples were diluted in Sample Diluent in accordance with the manufacturer’s instructions (ProteinSimple, CA, USA). Each sample was analyzed in a single well, as the Simple Plex microfluidic platform performs automatic triplicate measurements.

#### 2.2.2 Gene expression in PBMCs

Peripheral blood mononuclear cells were isolated by density gradient (Lympholyte-H, Cedarlane, Canada). Total RNA was extracted from PBMCs using the Chomczynski and Sacchi’s modified method (Chomczynski and Sacchi, 2006) and reverse-transcribed using the SuperScript VILOTM cDNA Synthesis Kit (Invitrogen by Thermo Fisher Scientific, Massachusetts, United States). Quantitative PCR (qPCR) was performed by OpenArray^®^ system QuantStudio 12K Flex Real-Time PCR System (Applied Biosystems by Thermo Fisher Scientific, Massachusetts, United States) using the commercial probes shown in Supplementary Table 1. The Glyceraldehyde-3-Phosphate Dehydrogenase (GAPDH), β-Actin (ACTB), and 18S Ribosomal RNA (18S) genes were included in the OpenArray^®^ chip and used as housekeeping endogenous control genes.

#### 2.2.3 Apolipoprotein E genotyping

The apolipoprotein E (APOE) genotype was determined using a PCR restriction fragment length polymorphism (PCR-RFLP) as previously described (Ferri et al., 2019).

### 2.3 Statistical analysis

The statistical analysis was conducted using IBM SPSS Statistic software (version 29, IBM Inc., Chicago, IL, USA). Since all continuous variables were non-normally distributed, they were expressed as median and interquartile range (IQR: 25–75th percentile) and analyzed with the Mann-Whitney U test. Categorical variables (i.e., sex, APOE genotype and education) were expressed as percentages and analyzed with Pearson’s Chi-squared test.

Regression analysis adjusted for log-transformed age, sex and presence of the APOE ε4 allele was used to assess the association between log-transformed gene expressions of HIF1A and HIF1AN and the AD risk (logistic analysis) and MMSE (linear analysis). Linear regression analysis adjusted for log-transformed age, sex, presence of the APOE ε4 allele, and diagnosis was used to assess the association between (i) the log-transformed gene expression of HIF1A or HIF1AN (independent variable) and the log-transformed gene expression and protein concentration of the neuroinflammatory markers analyzed (dependent variable), and (ii) the log-transformed gene expression of the neuroinflammatory marker (independent variable) and the log-transformed protein concentration of the same marker (dependent variable).

Values of *p* < 0.05 were considered statistically significant.

## 3 Results

Median age and sex distribution were similar in CT and AD (Table 2). As expected, MMSE, CDT, ADL, GDS, presence of the APOE ε4 allele and education were significantly different between CT and AD. The gene expressions of HIF1A and HIF1AN were significantly lower in AD than in CT (Table 2). Regarding inflammatory biomarkers, TNF-α gene expression was significantly reduced in PBMCs from AD patients, while TREM-1 gene expression showed a trend toward reduction compared with controls (Table 3). In plasma, as previously described (Arosio et al., 2025a), IL-6 and IL-10 concentrations were increased, whereas IL-1β concentration was decreased in AD patients than controls (Table 3).

**TABLE 2:** Characteristics of people categorized in controls and Alzheimer’s disease (AD).

Variables	Controls (*n* 308)	AD (*n* 83)	*p*
Age (years)	78.0 (73.0–82.0)	79.0 (76.0–81.0)	0.24
Sex (% women)	72.7%	71.1%	0.78
MMSE	29.0 (28.0–30.0)	21.0 (18.0–24.0)	**<0.001**
CDT	4.0 (3.0–5.0)	2.0 (0.0–4.0)	**<0.001**
ADL	6.0 (5.0–6.0)	5.0 (5.0–6.0)	**0.007**
GDS	6.0 (2.7–10.2)	8.0 (5.0–12.0)	**0.04**
APOE (% ε4 carriers)	19.9%	48.2%	**<0.001**
Education (%)			**<0.001**
0–5 years	17.7%	50.0%	
6–8 years	30.0%	22.7%	
9–13 years	30.4%	15.2%	
≥14 years	21.8%	12.1%	
HIF1A	1.22 (0.79–2.05)	0.86 (0.53–1.51)	**0.001**
HIF1AN	0.85 (0.70–1.39)	0.74 (0.58–1.10)	**0.008**

Age, MMSE, CDT, ADL, GDS, HIF1A and HIF1AN were expressed as median (interquartile range). Sex distribution was reported as the percentage of women. Genotype distribution of the APOE gene was reported as the percentage of carriers of at least one ε4 allele. Education was indicated as the percentage of subjects who received education for 0–5 years, 6–8 years, 9–13 years, and over 14 years. AD, Alzheimer’s disease; MMSE, Mini-Mental State Examination; CDT, Clock Drawing Test; ADL, activities of daily living; GDS, Geriatric Depression Scale; APOE, apolipoprotein E; HIF1A, hypoxia-inducible factor 1A; HIF1AN, hypoxia-inducible factor 1 alpha subunit suppressor. Bold values indicate statistically significant results (*p* < 0.05).

**TABLE 3:** Gene expression in PBMCs and plasma concentrations of inflammatory biomarkers in controls and patients with Alzheimer’s disease (AD).

Variables	Controls (*n* 308)	AD (*n* 83)	*p*
Gene expression			
IL-6	0.48 (0.19–1.22)	0.34 (0.12–1.21)	0.12
IL-10	0.53 (0.29–0.82)	0.51 (0.25–0.96)	0.96
TNF-α	0.39 (0.16–0.88)	0.21 (0.11–0.73)	**0.008**
IL-1B	0.46 (0.14–1.77)	0.39 (0.12–1.45)	0.55
TREM-1	0.97 (0.54–2.35)	0.83 (0.45–1.63)	**0.05**
Protein concentration			
IL-6 (pg/mL)	2.60 (1.73–4.06)	3.03 (1.98–5.37)	**0.04**
IL-10 (pg/mL)	2.04 (1.58–2.65)	2.55 (2.15–3.30)	**<0.001**
TNF-α (pg/mL)	9.01 (7.56–10.90)	9.22 (7.68–11.1)	0.79
IL-1β (pg/mL)	0.15 (0.08–0.26)	0.12 (0.04–0.21)	**0.02**
sTREM-1 (ng/mL)	0.48 (0.39–0.62)	0.49 (0.39–0.58)	0.97

Gene expression and plasmatic concentrations of the inflammatory biomarkers were expressed as median (interquartile range). AD, Alzheimer’s disease; IL-6, interleukin-6; IL-10, interleukin-10; TNF-α, tumour necrosis factor-α; IL-1B/IL-1β, interleukin-1β; TREM-1, triggering receptor expressed on myeloid cells-1; sTREM-1, soluble triggering receptor expressed on myeloid cells-1. Bold values indicate statistically significant results (*p* < 0.05).

Logistic regression analyses adjusted for age, sex and presence of the APOE ε4 allele showed that the low gene expressions of HIF1A (OR = 0.59, 95% CI = 0.42–0.83, *p* = 0.002) and HIF1AN (OR = 0.49, 95% CI = 0.30–0.82, *p* = 0.007) were associated with higher odds of AD.

Moreover, linear regression analyses adjusted for age, sex and presence of the APOE ε4 allele showed a positive association between the gene expressions of both HIF1A and HIF1AN and MMSE at diagnosis (HIF1A: R^2^ = 0.02, *B* = 0.48, SE = 0.19, *p* = 0.01; HIF1AN: R^2^ = 0.04, *B* = 0.45, SE = 0.12, *p* < 0.001).

In the overall cohort, results from multiple linear regression models [using the gene expressions and protein concentrations as dependent variables, and age, sex, APOE ε4 carrier status, diagnosis, and the gene expression of HIF1A (A) or HIF1AN (B) as independent variables] are reported in Tables 4, 5.

**TABLE 4:** Multiple linear regression model between the gene expression of each inflammatory biomarker (dependent variable) and age, sex (reference group: men), presence of the APOE ε4 allele (reference group: ε4 carriers), diagnosis (reference group: AD), and the gene expression of HIF1A (A) or HIF1AN (B) (independent variables).

Variables	A					B				
	Covariates	R^2^	B	SE	*p*	Covariates	R^2^	B	SE	*p*
IL-6	Age	0.07	−0.79	0.91	0.38	Age	0.04	−0.67	0.94	0.47
	Sex		−0.16	0.15	0.29	Sex		−0.13	0.15	0.39
	APOE		0.24	0.16	0.13	APOE		0.19	0.16	0.24
	Diagnosis		−0.24	0.17	0.17	Diagnosis		−0.40	0.17	**0.02**
	HIF1A		0.37	0.08	**<0.001**	HIF1AN		−0.40	0.13	**0.002**
IL-10	Age	0.03	0.96	0.59	0.11	Age	0.01	0.63	0.59	0.29
	Sex		−0.04	0.10	0.70	Sex		−0.04	0.10	0.68
	APOE		−0.09	0.10	0.40	APOE		−0.09	0.10	0.36
	Diagnosis		0.01	0.11	0.97	Diagnosis		−0.01	0.11	0.95
	HIF1A		0.15	0.05	**0.006**	HIF1AN		−0.08	0.09	0.38
TNF-α	Age	0.06	0.56	0.80	0.48	Age	0.03	0.40	0.81	0.62
	Sex		0.13	0.13	0.31	Sex		0.14	0.13	0.28
	APOE		0.07	0.14	0.59	APOE		0.09	0.14	0.52
	Diagnosis		−0.38	0.15	**0.04**	Diagnosis		−0.44	0.15	**0.004**
	HIF1A		0.26	0.07	**0.001**	HIF1AN		−0.08	0.11	0.50
IL-1B	Age	0.08	0.78	1.11	0.48	Age	0.09	0.50	1.10	0.65
	Sex		0.12	0.18	0.52	Sex		0.12	0.18	0.50
	APOE		0.25	0.19	0.18	APOE		0.25	0.19	0.18
	Diagnosis		−0.10	0.21	0.64	Diagnosis		−0.38	0.20	0.06
	HIF1A		0.52	0.10	**<0.001**	HIF1AN		−0.89	0.15	**<0.001**
TREM-1	Age	0.23	−0.59	0.56	0.29	Age	0.16	−0.69	0.58	0.23
	Sex		0.06	0.09	0.50	Sex		0.10	0.09	0.31
	APOE		0.06	0.10	0.56	APOE		−0.01	0.10	0.97
	Diagnosis		−0.04	0.11	0.67	Diagnosis		−0.37	0.11	**0.001**
	HIF1A		0.53	0.05	**<0.001**	HIF1AN		−0.64	0.08	**<0.001**

R^2^, coefficient of determination; B, unstandardized β coefficient; SE, standard error for the unstandardized β coefficient; APOE, apolipoprotein E; HIF1A, hypoxia-inducible factor 1 alpha subunit; HIF1AN, hypoxia-inducible factor 1 alpha subunit suppressor; IL-6, interleukin-6; IL-10, interleukin-10; TNF-α, tumour necrosis factor-α; IL-1B, interleukin-1β; TREM-1, triggering receptor expressed on myeloid cells-1. Bold values indicate statistically significant results (*p* < 0.05).

**TABLE 5:** Multiple linear regression model between the protein concentration of each inflammatory biomarker (dependent variable) and age, sex (reference group: men), presence of the APOE ε4 allele (reference group: ε4 carriers), diagnosis (reference group: AD), and the gene expression of HIF1A (A) or HIF1AN (B) (independent variables).

Variables	A					B				
	Covariates	R^2^	B	SE	*p*	Covariates	R^2^	B	SE	*p*
IL-6 (pg/mL)	Age	0.08	2.26	0.53	**<0.001**	Age	0.10	2.29	0.54	**<0.001**
	Sex		0.14	0.09	0.13	Sex		0.19	0.09	**0.03**
	APOE		−0.09	0.09	0.32	APOE		−0.10	0.09	0.27
	Diagnosis		0.20	0.11	0.06	Diagnosis		0.24	0.11	**0.03**
	HIF1A		0.01	0.05	0.82	HIF1AN		−0.13	0.08	0.08
IL-10 (pg/mL)	Age	0.09	1.42	0.37	**<0.001**	Age	0.11	1.46	0.36	**<0.001**
	Sex		0.17	0.06	**0.007**	Sex		0.17	0.06	**0.006**
	APOE		−0.01	0.06	0.88	APOE		−0.01	0.06	0.81
	Diagnosis		0.21	0.08	**0.007**	Diagnosis		0.19	0.07	**0.007**
	HIF1A		−0.02	0.03	0.61	HIF1AN		−0.12	0.05	**0.02**
TNF-α (pg/mL)	Age	0.11	1.05	0.20	**<0.001**	Age	0.11	1.13	0.20	**<0.001**
	Sex		0.06	0.03	0.07	Sex		0.08	0.03	**0.02**
	APOE		−0.04	0.03	0.29	APOE		−0.06	0.04	0.09
	Diagnosis		0.05	0.04	0.24	Diagnosis		0.03	0.04	0.49
	HIF1A		0.03	0.02	0.07	HIF1AN		0.02	0.03	0.49
IL-1β (pg/mL)	Age	0.08	0.38	0.74	0.61	Age	0.03	0.45	0.75	0.55
	Sex		0.05	0.12	0.67	Sex		0.03	0.12	0.79
	APOE		−0.21	0.13	0.11	APOE		−0.23	0.13	0.08
	Diagnosis		−0.20	0.15	0.18	Diagnosis		−0.32	0.15	**0.03**
	HIF1A		0.30	0.07	**<0.001**	HIF1AN		0.02	0.10	0.86
sTREM-1 (pg/mL)	Age	0.13	1.30	0.25	**<0.001**	Age	0.12	1.33	0.25	**<0.001**
	Sex		0.10	0.04	**0.02**	Sex		0.11	0.04	**0.007**
	APOE		−0.09	0.04	**0.03**	APOE		−0.10	0.04	**0.02**
	Diagnosis		0.06	0.05	0.21	Diagnosis		0.02	0.05	0.69
	HIF1A		0.05	0.02	**0.02**	HIF1AN		−0.01	0.03	0.78

R^2^, coefficient of determination; B, unstandardized β coefficient; SE, standard error for the unstandardized β coefficient; APOE, apolipoprotein E; HIF1A, hypoxia-inducible factor 1 alpha subunit; HIF1AN, hypoxia-inducible factor 1 alpha subunit suppressor; IL-6, interleukin-6; IL-10, interleukin-10; TNF-α, tumour necrosis factor-α; IL-1β, interleukin-1β; sTREM-1, soluble triggering receptor expressed on myeloid cells-1. Bold values indicate statistically significant results (*p* < 0.05).

In the multiple linear regression models shown in Table 4, we observed that the gene expressions of IL-6, IL-10, TNF-α, IL-1B, and TREM-1 were positively associated with the gene expression of HIF1A (Table 4A), while IL-6, IL-1B, and TREM-1 were negatively associated with the gene expression of HIF1AN (Table 4B). TNF-α gene expression was associated with diagnosis in both the HIF1A and HIF1AN models (Tables 4A, B), while IL-6 and TREM-1 gene expressions were associated with diagnosis only in the HIF1AN model (Table 4B).

With regard to plasma concentrations, IL-1β and sTREM-1 were positively associated with HIF1A gene expression (Table 5A), while IL-10 showed a negative association with HIF1AN gene expression (Table 5B). In both the HIF1A and HIF1AN models, IL-6 and TNF-α concentrations were positively associated with age, while IL-10 and sTREM-1 concentrations were positively associated with both age and sex (Tables 5A, B). Furthermore, IL-6 and TNF-α concentrations were associated with sex only in the HIF1AN model (Table 5B). Additionally, IL-10 concentrations were associated with diagnosis in both the HIF1A and HIF1AN models (Tables 5A, B), whereas IL-6 and IL-1β only in the HIF1AN model (Table 5B). Finally, sTREM-1 was associated with APOE ε4 carrier status in both models (Tables 5A, B).

Interestingly, in linear regression analysis adjusted for age, sex, APOE ε4 carrier status, and diagnosis, TREM-1 gene expression showed a positive association with sTREM-1 plasma concentrations (R^2^ = 0.14, *B* = 0.05, SE = 0.02, *p* = 0.01). No other significant associations between gene expressions and protein concentrations were found for the other markers analyzed.

## 4 Discussion

To our knowledge, this is the first study that described lower expression of the HIF1A and HIF1AN genes in PBMCs from AD compared to gender- and age-matched subjects without cognitive decline. Moreover, lower gene expressions of both genes were found to be associated with higher odds of AD and, consequently, with low MMSE values. This suggests that these genes may be involved not only in AD-related pathological processes, but also in the clinical manifestations of the disease (Mitroshina and Vedunova, 2024).

Analysis of HRE-containing inflammatory markers revealed significantly reduced TNF-α gene expression and a decreasing trend in TREM-1 gene expression in AD patients compared with controls. Furthermore, plasma IL-6 and IL-10 concentrations were significantly increased, whereas IL-1β concentration was significantly reduced in AD patients than controls.

Interestingly, in PBMCs from the overall cohort, the gene expression of HIF1A was positively associated with the gene expressions of the analyzed inflammatory markers (IL-6, IL-10, TNF-α, IL-1β, and TREM-1), after adjustment for all covariates. On contrary, HIF1AN gene expression was significantly and negatively associated with the gene expressions of IL-6, IL-1β, and TREM-1. Finally, HIF1A gene expression was positively associated only with the plasmatic concentrations of IL-1β and sTREM-1, whereas HIF1AN gene expression was negatively associated only with IL-10 plasmatic concentrations. Therefore, as expected, our findings indicate a more consistent pattern of associations between HIF1A and HIF1AN gene expressions and the analyzed inflammatory markers at the gene expression level, in line with the role of HIF-1α as a transcription factor. In contrast, fewer associations were observed with the corresponding circulating inflammatory marker concentrations, a discrepancy likely explained by multiple regulatory mechanisms acting both at post-transcriptional and post-translational levels (Du et al., 2014; Uchida et al., 2019).

During aging, the ability to adapt to hypoxia is reduced (Catchlove et al., 2018; Salvatore et al., 2022; Bennett et al., 2024), increasing both susceptibility to diseases and the rate of their course (Ogunshola and Antoniou, 2009). A recent model of dementia dynamics proposes damage to cerebral vasculature as an early event in the dementia continuum typical of AD (Sargurupremraj et al., 2020; Rajeev et al., 2022). In this regard, dysregulation of the HIF-1 pathway may play a central role in this pathophysiological process by impairing cellular responsiveness to hypoxia, promoting neurovascular injury and exacerbating inflammatory responses (McGettrick and O’Neill, 2020; Sargurupremraj et al., 2020; Wang et al., 2021; Rajeev et al., 2022).

This is supported by the reduced gene expressions of HIF1A and HIF1AN in PBMCs from our AD cohort, and by the finding that, after adjustment for all covariates, their expression levels were significantly associated not only with the expression of inflammatory components but also with the presence of AD. Taken together, these findings suggest that altered HIF-1α signaling may contribute to impaired cellular homeostasis in these peripheral immune cells, potentially affecting their adaptive responses even in the absence of a real hypoxic stimulus.

The significant reduction in TNF-α gene expression, together with a trend toward decreased TREM-1 gene expression in PBMCs from AD patients compared to controls, further supports peripheral immune dysregulation in AD. Güven et al. (2024) reported downregulation of both TNF-α and IL-6 gene expressions in PBMCs from AD patients, suggesting impaired peripheral immune responsiveness. However, we did not observe significant differences in IL-6, IL-10 or IL-1β gene expressions between AD and controls, consistent with the heterogeneous findings reported in the literature (Xiong et al., 2021; Xu and Jia, 2021). Furthermore, although increased TREM-1 gene expression has been described in peripheral leukocytes from AD patients (Sao et al., 2018), we observed a decreasing trend in our AD patients. Taken together, these discrepancies likely reflect the complexity of peripheral immune responses in AD, which may vary according to age and disease stage.

Notably, HIF-1α activation appears to be linked to neuroinflammation, a key feature of AD, promoting the expression of inflammatory cytokines and exacerbating neuronal damage (Porel et al., 2025). Furthermore, in the brain, dysregulated HIF-1α signaling seems to contribute to microglial activation and astrocytic reactivity (Porel et al., 2025).

In PBMCs from our cohort, the positive associations observed between HIF1A gene expression and the gene expressions of IL-6, IL-10, TNF-α, IL-1β and TREM-1, together with the negative associations between HIF1AN gene expression and the gene expressions of IL-6, IL-1β and TREM-1, further support the role for HIF-1α signaling in inflammatory responses (Corcoran and O’Neill, 2016; McGettrick and O’Neill, 2020). HIF-1α is a key transcription factor in immune cells, where it regulates both pro- and anti-inflammatory cytokine expression by binding to HREs within the promoters of its target genes (Corcoran and O’Neill, 2016; McGettrick and O’Neill, 2020; Malkov et al., 2021; Tang et al., 2022; Lampiasi and Russo, 2026). Accordingly, the positive association of HIF1A and the negative association of HIF1AN gene expressions with IL-6, IL-1B, and TREM-1 gene expressions are consistent with a key contribution of HIF-1α signaling to the transcriptional regulation of these pro-inflammatory genes via HRE binding in PBMCs from our cohort.

Conversely, the positive association between HIF1A gene expression and IL-10 and TNF-α gene expression, in the absence of a corresponding association with HIF1AN gene expression, could indicate that additional regulatory mechanisms may contribute to the modulation of the expression of these cytokines. Notably, HIF-1α has been reported to enhance the transcription of inflammatory cytokines also through crosstalk with nuclear factor kappa-light-chain-enhancer of activated B cells (NF-κB) (Tannahill et al., 2013; Lampiasi and Russo, 2026), the main transcription factor regulating inflammation (Anilkumar and Wright-Jin, 2024; Mao et al., 2025). Indeed, under hypoxic condition, HIF-1α and NF-κB signaling pathways are tightly interconnected in a bidirectional manner, as NF-κB can transcriptionally induce HIF1A gene expression and stabilize HIF-1α, while HIF-1α, in turn, interacts with NF-κB and enhance its activity to promote inflammatory responses (Cummins et al., 2006; Taylor, 2008; Lampiasi and Russo, 2026).

Overall, our data indicate a close link between HIF-1α signaling and inflammatory responses at the transcriptional level in circulating immune cells, supporting the evidence that, beyond its canonical role as a hypoxia-responsive factor, HIF-1α may contribute to immunometabolic adaptation (McGettrick and O’Neill, 2020; Porel et al., 2025) that characterizes the AD etiopathogenesis. In this context, HIF-1α–related transcriptional activity may contribute to systemic inflammatory processes relevant to aging and AD (Franceschi et al., 2017; Sargurupremraj et al., 2020).

As expected, these findings were only partially reflected in the plasmatic concentrations of the inflammatory components, with only the positive associations between HIF1A gene expression and plasma IL-1β and TREM-1 concentrations confirmed, possibly reflecting a stronger and more direct transcriptional influence of HIF-1α on these specific markers, as previously suggested in models of myeloid cell activation (Ting et al., 2024).

These observations are consistent with previous studies showing that HIF-1α and IL-1β are functionally interconnected, with evidence of reciprocal regulation of their signaling pathways (Lampiasi and Russo, 2026). In mouse macrophages exposed to hypoxia, inflammatory-like stimuli, such as lipopolysaccharide, induce HIF-1α stabilization and promote IL-1β production, which is markedly reduced upon HIF-1α inhibition, suggesting that HIF-1α may directly regulate the increase in IL-1β production (Tannahill et al., 2013). On the other hand, IL-1β can enhance HIF-1α transcription and stabilization even under normoxic conditions (Rius et al., 2008; Tannahill et al., 2013; Palazon et al., 2014). Moreover, it was also observed that endoplasmic reticulum stress-associated pathways may synergize with HIF-1α in immune cells as an adaptive response to oxygen deprivation, promoting cytokine secretion, including IL-1β (Mawambo et al., 2023). Several cytokines, including IL-1β, promote microglial activation and mediate amyloid-β (Aβ) clearance (Shaftel et al., 2007; Fu et al., 2016), suggesting the presence of an initially sustained inflammatory response during the early phases of neurodegeneration. This response is thought to decline in overt dementia (Arosio et al., 2025a), possibly reflecting exhaustion of its neuroprotective component and consistent with the reduced plasma IL-1β concentrations observed in our AD cohort.

The increased plasma concentrations of IL-6 and IL-10 in AD patients compared with controls are in line with the well-established concept that persistent activation of pro-inflammatory pathways, coupled with an insufficient anti-inflammatory response, represents a hallmark of AD, as we have previously reported in a cohort of older patients with dementia (Arosio et al., 2025a). This pattern is consistent with the dualistic role of inflammation in AD, which may be either protective or detrimental depending on the biological stage of the disease, and with previous evidence showing that FIH-dependent modulation of HIF-1α activity influences inflammatory cytokine profiles in myeloid cells (Schützhold et al., 2022; Ting et al., 2024).

Consistent with the immunological alterations observed in our model, both TREM-1 gene expression and plasma concentrations were significantly and positively associated with HIF1A gene expression. This finding supports previous evidence that TREM-1 is transcriptionally regulated by HIF-1α via HRE in its promoter region (Bosco et al., 2011; Dong et al., 2024). TREM-1 is a transmembrane receptor expressed by innate immune cells, known to act synergistically with toll-like receptors to amplify inflammatory responses to pathogens, contributing to sepsis-induced immune dysregulation and organ dysfunction (Bouchon et al., 2001; Jolly et al., 2018; Boufenzer et al., 2021). Although TREM-1 has been primarily linked to infectious diseases (Bouchon et al., 2001), its involvement in the pathophysiology of non-infectious conditions, including neuroinflammatory and neurodegenerative disorders, is increasingly recognized (Gibot et al., 2004a,b; Barraud and Gibot, 2011; Tammaro et al., 2017; Li et al., 2025).

Its soluble form (sTREM-1), which reflects TREM-1 receptor processing, is released following proteolytic cleavage of the membrane-bound receptor upon cellular activation during acute inflammatory responses and tissue injury, and is widely recognized as a biomarker of disease severity and clinical outcomes (Jolly et al., 2021). Notably, sTREM-1 was the only marker associated with APOE ε4 status, a major genetic risk factor for AD, possibly reflecting the role of this receptor in cholesterol metabolism and lipid signaling in microglia as well as in periphery, similar to the well-established role for TREM-2 (Yin, 2023). Furthermore, sTREM-1 was the only marker showing a significant positive association between gene expression in PBMCs and plasma concentrations, whereas no such associations were observed for the other cytokines. This decoupling between gene expression and circulating levels is not unexpected for cytokines, which are tightly regulated at post-transcriptional and post-translational levels and have a short half-life due to rapid secretion and clearance (Du et al., 2014; Uchida et al., 2019). However, although sTREM-1 also has a short half-life, its circulating concentrations inherently reflect ongoing transcriptional activity, because its release via proteolytic cleavage depends strictly on the expression and activation of the TREM-1 receptor, making sTREM-1 a reliable marker of TREM-1 activation in innate immune cells (Jolly et al., 2021).

As expected, plasma concentrations of our inflammatory markers were significantly associated with age (Arosio et al., 2023), sex (Arosio et al., 2025b) and the presence of dementia (Roveta et al., 2024; Tylutka et al., 2024).

The main limitation of this study lies in its cross-sectional nature, which raises the possibility that the observed results obtained may reflect unmeasured lifestyle factors beyond the health determinants considered in the analyses. Although major potential confounders, such as age and sex, were taken into account, residual confounding cannot be fully excluded. Moreover, the absence of protein-level measurements in PBMCs represents an additional limitation, as it prevents direct validation of the observed transcriptional changes at the functional level. Finally, although PBMCs provide a useful peripheral model for studying AD-related alterations, the lack of direct measurements in the central nervous system and of functional assays limits the ability to fully elucidate the relationship between peripheral changes and brain-related mechanisms.

Overall, our findings propose an intriguing peripheral model that may reflect key pathogenic processes relevant to AD (Arosio et al., 2014; Phongpreecha et al., 2020; Costa et al., 2022; Dover et al., 2024), in which HIF1A gene expression is associated with the expression of neuroinflammation-related genes. In particular, the significantly reduced gene expression of both HIF1A and its negative regulator HIF1AN in PBMCs from AD patients may indicate an altered regulation of HIF-1α signaling in these cells, potentially recapitulating interesting pathogenic features of the disease even in the absence of a real hypoxic stimulus (Janoshazi et al., 2006; Marksteiner and Humpel, 2009; Wang et al., 2014; Hugon et al., 2018). Therefore, our findings provide further support for considering AD also a systemic disorder with manifestations extending beyond the central nervous system (Morris et al., 2014) and PBMCs as a window reflecting brain-related changes that involve both neuroinflammatory processes and hypoxia-related mechanisms (Sargurupremraj et al., 2020; Rajeev et al., 2022; Porel et al., 2025).

In conclusion, understanding the role of HIF-1α in AD provides valuable insights into disease mechanisms and offers new avenues for therapeutic intervention. Modulating HIF-1α activity may help mitigate key pathological features of the disease, including neuroinflammation. However, further studies are needed to elucidate the precise mechanisms underlying HIF-1α dysregulation in the dementia continuum typical of AD and to develop effective targeted therapies.

## Data Availability

The datasets presented in this article are not readily available due to privacy and ethical restrictions. Requests to access the datasets should be directed to the corresponding author, beatrice.arosio@unimi.it.
