# Supplementary material for: Unraveling the role of HIF-1 in peripheral blood mononuclear cells from older patients with Alzheimer’s disease
Source: Front Aging Neurosci. 2026 Jun 3;18:1831919. doi: 10.3389/fnagi.2026.1831919 (PMC13272122; doi:10.3389/fnagi.2026.1831919)
Supplement: Supplementary file 1 [file Table_1.docx]

**Supplementary Table 1.** List of genes with corresponding commercially available probes used for qPCR analysis.

| **Gene** | **Description** | **Probe** |
| --- | --- | --- |
| HIF1A | Hypoxia Inducible Factor 1 Subunit Alpha | Hs00936371_m1 |
| HIF1AN | Hypoxia Inducible Factor 1 Subunit Alpha Inhibitor | Hs00215495_m1 |
| IL-6 | Interleukin-6 | Hs00174131_m1 |
| IL-10 | Interleukin-10 | Hs00961622_m1 |
| TNF-α | Tumour necrosis factor-α | [Hs00174128_m1](https://www.thermofisher.com/taqman-gene-expression/product/Hs00174128_m1?CID=&ICID=&subtype=ge_all) |
| IL-1B | Interleukin-1β | [Hs01555410_m1](https://www.thermofisher.com/taqman-gene-expression/product/Hs01555410_m1?CID=&ICID=&subtype=ge_all) |
| TREM-1 | Triggering Receptor Expressed on Myeloid Cells 1 | Hs00218624_m1 |
| GAPDH | Glyceraldehyde-3-Phosphate Dehydrogenase | Hs99999905_m1 |
| ACTB | β-Actin | Hs99999903_m1 |
| 18S | 18S Ribosomal RNA | Hs99999901_s1 |
|  |  |  |
|  |  |  |
|  |  |  |
|  |  |  |
|  |  |  |
|  |  |  |
